# Estrogen Promotes Hepatic Synthesis of Long-Chain Polyunsaturated Fatty Acids by Regulating *ELOVL5* at Post-Transcriptional Level in Laying Hens

**DOI:** 10.3390/ijms18071405

**Published:** 2017-06-30

**Authors:** Meng Zhang, Cui-Cui Li, Fang Li, Hong Li, Xiao-Jun Liu, Juan J. Loor, Xiang-Tao Kang, Gui-Rong Sun

**Affiliations:** 1 College of Animal Science and Veterinary Medicine, Henan Agricultural University, Zhengzhou 450002, China; zm19911213@gmail.com (M.Z.); lcc19900219@gmail.com (C.-C.L.); lf19920909@gmail.com (F.L.); hl2017@henau.edu.cn (H.L); Xjliu2008@hotmail.com (X.-J.L.); xtkang2001@263.net (X.-T.K.); 2 Henan Innovative Engineering Research Center of Poultry Germplasm Resource, Zhengzhou 450002, China; 3 Department of Animal Sciences and Division of Nutritional Sciences, University of Illinois, Urbana, IL 61801, USA; jloor@illinois.edu

**Keywords:** chicken, *ELOVL5*, liver, estrogen, miRNA, LCPUFA

## Abstract

The very long chain fatty acid elongase (ELOVL) plays an important role in the synthesis of long-chain polyunsaturated fatty acids (LCPUFA). Previous studies suggest that chicken could be an alternate source of eicosapentaenoic acid (EPA) and docosahexaenoic acid (DHA). In this study, we detected that *ELOVL5*, which plays a key role in the biosynthesis of omega-3 (*n*-3) and omega-6 (*n*-6) polyunsaturated fatty acids (PUFA), was highly expressed in the liver of laying hens and increased rapidly after sexual maturity. Bioinformatic analysis revealed ELOVL fatty acid elongase 5 (*ELOVL5*) gene as a putative target of miR-218-5p, miR-19a-3p, miR-19b-3p, miR-30a-5p, miR-30b-5p, and miR-30e-5p. We demonstrated estrogen downregulated microRNA (miRNA), and that *ELOVL5* is a direct target of miR-218-5p, which was located in intron 14 of the Slit guidance ligand 2 (*SLIT2*) gene and co-expressed with the host gene. Overall, estrogen enhanced hepatic synthesis of LCPUFA by functioning as a negative regulator of miRNA thereby augmenting the expression of these miRNA target genes, especially *ELOVL5*, which plays a key role in the biosynthesis of *n*-3 and *n*-6 LCPUFA. This study provides a novel model for the use of estrogen in the poultry industry as an inducer of *ELOVL5* expression to enhance hepatic *n*-3 and *n*-6 LCPUFA synthesis at the post-transcriptional level.

## 1. Introduction

Fish and fish oil are the most common dietary sources of omega-3 (*n*-3) long-chain polyunsaturated fatty acids (LCPUFA), namely eicosapentaenoic acid (EPA) and docosahexaenoic acid (DHA) [1,2,3]. The beneficial health effects of EPA and DHA have driven human consumption of marine fauna to an extent where there are concerns about the future ability of aquaculture to meet this demand [4,5]. Recent studies have suggested that chicken could be able to synthesize more docosapentaenoic acid (DPA) than other species through 24:5*n*-3, the precursor of DHA [1,4,6]. The enzymatic elongation of very long-chain fatty acids (*ELOVL5*), which is encoded as *ELOVL5*, plays a key role in the biosynthesis of *n*-3 and omega-6 (*n*-6) polyunsaturated fatty acids (PUFA) [7]. Different from mammals and most fish, chicken *ELOVL5* can elongate DPA to 24:5*n*-3 [1]. Previous studies suggested that compared with other species, the chicken *ELOVL5* enzyme has a five-fold conversion rate from DPA (22:5*n*-3) to 24:5*n*-3, which is the penultimate precursor of DHA [1,6]. The fatty acids (FA) synthesis in birds occurs mainly in the liver [8]. In addition, the liver plays a central role in whole-body lipid metabolism, which encompasses the synthesis and modification of fatty acids by way of desaturation, elongation, and oxidation [7]. These key enzymes of fatty acid metabolism are highly expressed in the liver, and their activities and expression are regulated during development by diet, hormones, and other factors, in mature animals [9]. Many studies have focused on the dietary conversion of α-linolenic acid (ALA) and the substrate specificity of *ELOVL5* in the synthesis of *n*–3 LCPUFA [1,4,6,10]. However, little focus has been placed on the regulatory function of other factors such as hormones and non-coding RNAs.

Estrogen is vitally important for sexual maturity and the development of the female reproductive system [11,12]. It is generally believed that the estrogen level of hens rises significantly during the egg-laying period, as do many of the genes and their products involved in hepatic lipid and fatty acid metabolism in liver tissue [13,14,15]. Our previous liver transcriptome research in laying hens showed that *ELOVL5* increased by 1.98-fold in peak-laying-period hens (30 weeks old) compared with pre-laying-period hens (20 weeks old) [16]. An increasing number of studies have demonstrated that miRNA serve as important regulators in lipid and fatty acid metabolism [11,12,13,14]. Our recent study revealed several differently expressed miRNA in liver between pre- and peak-laying-period hens, which suggested that hepatic miRNA might play a role in the regulation of hepatic lipid and fatty acid synthesis [17].

To investigate the effect of estradiol on the synthesis of long-chain polyunsaturated fatty acids, we focused on *ELOVL5*, which plays a key role in the biosynthesis of PUFA. In the present study, we found that *ELOVL5* was highly expressed in liver tissue and increased rapidly after sexual maturity. Combined with our previous study, we found six miRNA were significantly downregulated in peak-laying-period hens and primary hepatocytes treated with estrogen. Furthermore, we observed that miR-218-5p could directly target *ELOVL5*. Interestingly, we also found that miRNA-218-5p was co-expressed with the host gene *SLIT2*, which was suppressed by estrogen. Our results revealed that estrogen promotes the synthesis of LCPUFA indirectly through up-regulation of *ELOVL5* in the liver of laying hens. These findings provide a novel mechanism whereby estradiol increased *ELOVL5* expression to accelerate LCPUFA synthesis in the liver of laying hens.

## 2. Results

### 2.1. ELOVL5 Expression Rapidly Increased in Liver Tissue During Peak-Laying Stages

**Figure 1:**
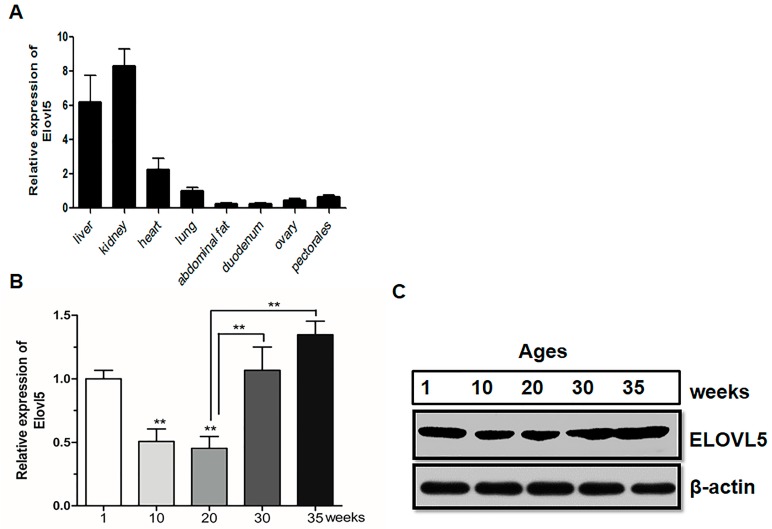
*ELOVL5* was highly expressed in liver tissue and increased rapidly during the peak-laying period. (**A**) Tissue expression profile of *ELOVL5* in laying hens; (**B**) Expression patterns of chicken *ELOVL5* in liver at different developmental stages. *β-Actin* was used as the referenced gene to estimate mRNA. Data are presented as Mean ± SEM (*n* = 6 for each group), * *p* < 0.05, ** *p* < 0.01; (**C**) Western blotting of *ELOVL5* using equal amounts of protein extracted from livers of chickens with different ages.

To detect the tissue-specific expression patterns of *ELOVL5* mRNA in chickens, tissue including liver, heart, lung, kidney, duodenum, ovary, pectorals, and abdominal fat from 30-week-old chickens were analyzed using qPCR. Our results showed that *ELOVL5* was highly expressed in liver and kidney tissues (Figure 1A). To investigate the expression patterns of *ELOVL5* in chicken liver, both mRNA and the protein level of *ELOVL5* at different developmental stages were analyzed. The results showed that *ELOVL5* was greatly increased after sexual maturity (Figure 1B,C) (*p* < 0.05).

### 2.2. Computational Prediction of the MicroRNAs Targeting ELOVL5

**Figure 2:**
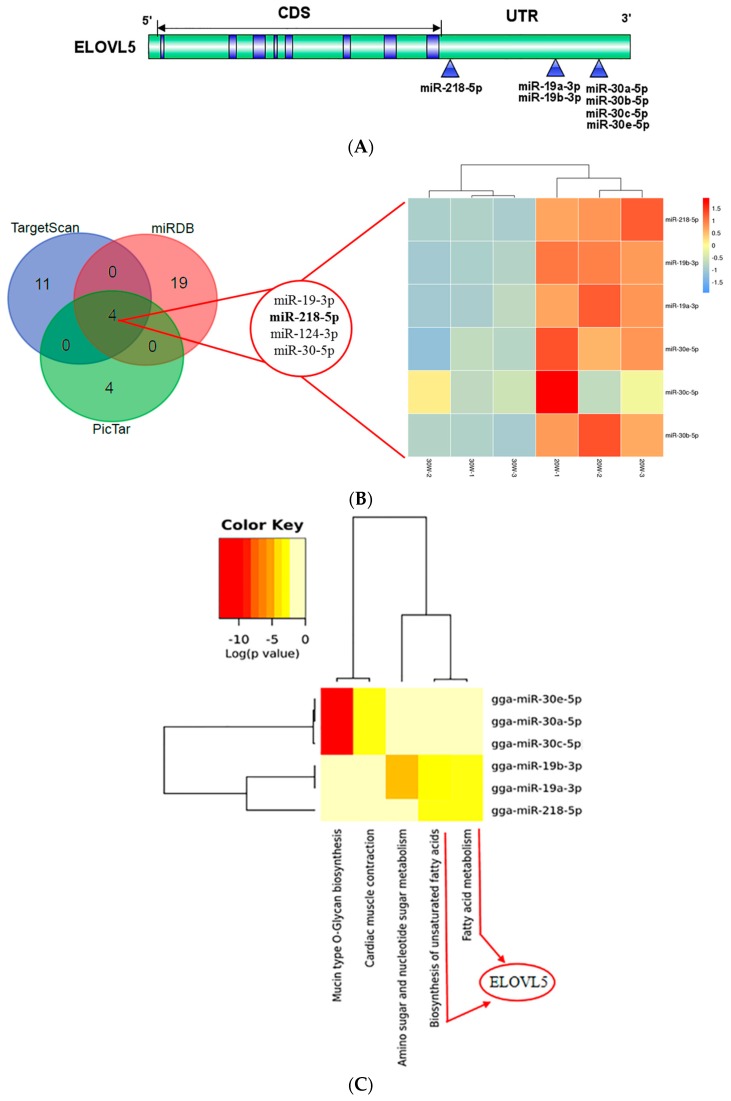
Integrated prediction of the miRNA targeted *ELOVL5*. (**A**) The binding sites between miRNA and 3′UTR. CDS, Coding Sequence; UTR, Untranslated Region; (**B**) TargetScan, miRDB and PicTar software analysis of miRNA that potentially target *ELOVL5*. The intersection of our previous miRNA data [17] and the predicted miRNA; Intronic miRNA is in bold. (**C**) Heat map of putative pathways of candidate miRNA by DIANA miRPath analysis.

By sequence comparison analysis, no putative estrogen receptor binding site upstream of the *ELOVL5* promoter was found in the present study(data not shown). To explore the underlying mechanism coordinated by estrogen, we focused on hepatic miRNAs that might target *ELOVL5* genes, and hence might affect the metabolism of fatty acids. Three miRNA target prediction tools, TargetScan, miRDB, and PicTar were used to search for the miRNA targeting *ELOVL5*. Two miRNA (miR-124a-3p and miR-124b) and four miRNA clusters (miR-19-3p, miR-218-5p, miR-124-3p, and miR-30-5p) shared by three databases were found to be potentially combine the 3′UTR of *ELOVL5*, respectively (Figure 2A,B and Figure S3). However, neither miR-124a-3p nor miR-124b were differentially expressed miRNA in our previous miRNA experiment [17]. To further classify and analyze the potential functions of these downregulated miRNA in peak laying period, we used DIANA mirPath to investigate the classification and their target pathways (Figure 2C). Among these pathways, the biosynthesis of unsaturated fatty acids (*p* < 0.01) and fatty acid metabolism (*p* < 0.05) signaling pathways were found to be involved in the target pathways (Figure 2C). According to our previous study [17], 46 differentially downregulated miRNA (|fold-change| > 1.5, FDR < 0.05) were re-analyzed to identify miRNA shared by the potential binding sites in *ELOVL5* (Table S1 and Figure 2B). Three miRNA clusters (miR-218-5p, miR-19-3p, and miR-30-5p) from 46 differentially expressed miRNA were found to potentially bind to the 3′UTR of *ELOVL5* in the present study (Figure 2B).

### 2.3. Estrogen Suppression of miRNA Expression to Upregulate the ELOVL5 Expression in Chicken Hepatocytes

**Figure 3:**
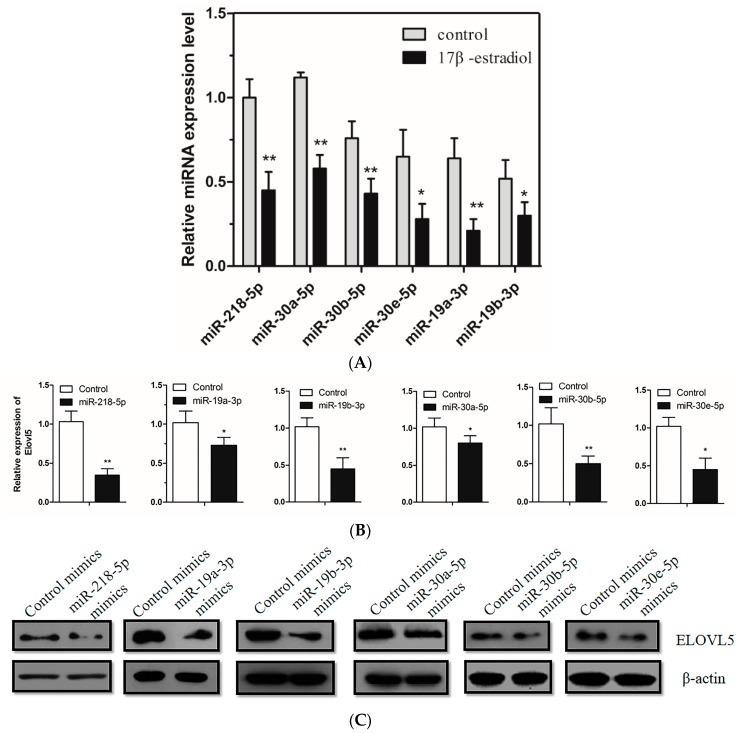
Estrogen suppresses miRNA expression to upregulate the expression of *ELOVL5* in chicken hepatocytes. (**A**) Effect of estrogen on miRNA expression in hepatocytes. miRNA expression levels were detected by qPCR in hepatocytes incubated for 24 h with 100 nM 17 β-estradiol. *U6* was used as the reference gene to estimate miRNA relative expression. Data represent Mean ± SEM (*n* = 6) of miRNA expression relative to miR-218-5p level in vehicle group, set to a value of 1. * *p* < 0.05; ** *p* < 0.01; (**B**) miRNA suppresses the expression of *ELOVL5* in hepatocytes. Mimic miRNA were used to overexpress miRNA (miR-218-5p, miR-19a-3p, miR-19b-3p, miR-30a-5p, miR-30b-5p, miR-30e-5p) in hepatocytes, with negative mimics as control group (*n* = 3). RNA were extracted 48 h later, and levels were detected by qPCR and western blot. * *p* < 0.05; ** *p* < 0.01; (**C**) Immunoblot analysis of *ELOVL5* in hepatocytes treated with control and miRNA mimics.

In order to understand the effect of estrogen on miRNA expression, miRNA expression level was measured by qPCR in hepatocytes incubated for 24 h with 100 nM 17 β-estradiol. As shown in Figure 3A, miR-218-5p, miR-30a-5p, miR-30b-5p, miR-30e-5p, miR-19a-3p, and miR-19b-3p were significantly downregulated in 17 β-estradiol treated groups (*p* < 0.01). Furthermore, a marked downregulation of *ELOVL5* mRNA and protein expression level were observed by overexpressing these miRNA in chicken primary hepatocytes (Figure 3B,C) (*p* < 0.01).

### 2.4. Verification of the Interaction between miR-218-5p and ELOVL5

**Figure 4:**
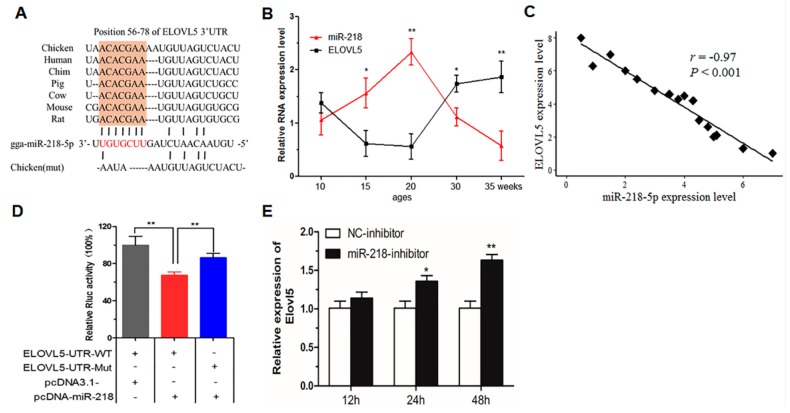
Identification of ELOVL5 as a direct target of miR-218-5p. (**A**) The putative miR-218-5p binding site at ELOVL5 3′UTR (red) is evolutionarily conserved across species. The shaded portion represents the seed region of miR-218-5p; (**B**) Expression levels of miR-218-5p (red) and ELOVL5 (black) mRNA in chicken liver at different developmental stages. The former was normalized to U6, while the latter to β-Actin. Data are presented as Mean ± SEM (*n* = 3 for each group); (**C**) The correlation between expression level of miR-218-5p and ELOVL5 in 16 liver tissue samples; (**D**) miR-218-5p suppresses ELOVL5 translation. pcDNA-miR-218 was transfected into DF1 cells along with ELOVL5-3′UTR-WT but not ELOVL5-3′UTR-Mut; (**E**) Negative control inhibitor (NC inhibitor) or miR-218-5p inhibitor was transfected into chicken primary hepatocytes. After 12, 24 and 48 h, qPCR assays were performed to determine the expression level of ELOVL5. RNA were extracted 48 h later, and levels were accessed by qPCR. The data represent Mean ± SEM (*n* = 6). * *p* < 0.05; ** *p* < 0.01.

The putative miR-218-5p-binding sites at *ELOVL5* 3′UTR are evolutionarily conserved across species (Figure 4A). To evaluate the possibility that *ELOVL5* expression is negatively regulated by candidate miRNA under normal physiological conditions, miR-218-5p and *ELOVL5* mRNA in chicken liver at different developmental stages were detected by qPCR. The results showed that the expression of miR-218-5p increased before 20 weeks of age and decreased after 20 weeks of age, whereas that of *ELOVL5* mRNA tended to have the opposite change (Figure 4B). The correlation coefficient between miR-218-5p and *ELOVL5* mRNA in chicken liver at different developmental stages was −0.906 (*p* = 0.034) in the present study (data not shown). In addition, the expression level of miR-218-5p and *ELOVL5* were measured in 16 liver tissue samples at peak- and later laying-period (35–55 weeks old). Results indicated that miR-218-5p was inversely correlated with *ELOVL5* in laying hens (*r* = −0.97, *p* < 0.001) (Figure 4C). To verify the direct binding between miR-218-5p and *ELOVL5*, a 3′UTR fragment with seed region binding site was inserted into the 3′UTR of a Renilla luciferase (*Rluc*) gene of the psiCHECK-2 vector (Figure 4A). Luciferase assay revealed that miR-218-5p significantly reduced the Rluc activity of the wild-type *ELOVL5* reporter vector (*ELOVL5*-UTR-WT) in DF1 cells, whereas point mutations of target sites bound by the seed region of miR-218-5p in the 3′UTR of *ELOVL5* did not disturb luciferase activity (Figure 4D). Overexpression of miR-218-5p in chicken hepatocytes significantly decreased *ELOVL5* mRNA and protein level (*p* < 0.05) (Figure 3A,B and Figure 4E). In contrast, miR-218-5p-inhibitor increased *ELOVL5* expression at 12, 24, and 48 h (Figure 4E). These data suggest the possibility that miR-218-5p negatively regulates the expression of *ELOVL5* in chicken liver.

### 2.5. Host Gene SLIT2 Inhibition by Estrogen and Co-Expressed with miR-218-5p

**Figure 5:**
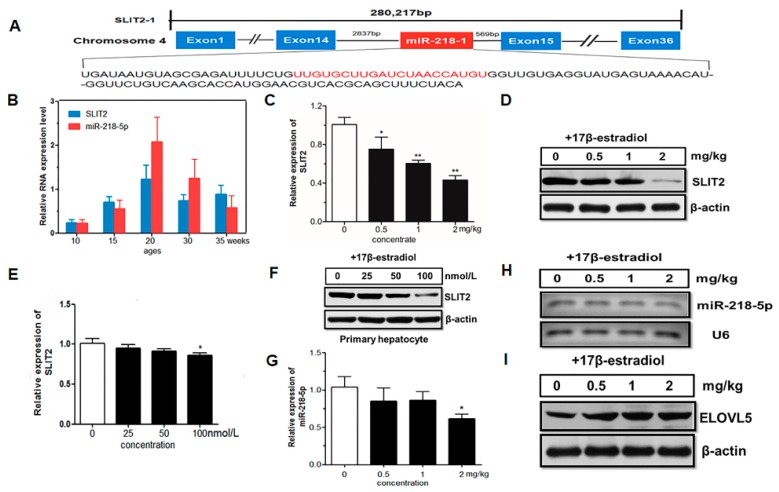
Intronic miR-218 is co-expressed with host gene *SLIT2*, which is downregulated by estrogen. (**A**) Chicken *SLIT2* harbors a related intronic miR-218-1. The sequences encoding the pre-miRNA are shown, with the mature miRNA sequences highlighted in red; (**B**) The expression levels of miR-218-5p and *SLIT2* mRNA in chicken liver at different developmental stages; (**C**,**D**) qPCR analysis and Western blot analysis of host gene *SLI T2* mRNA (**C**) and protein expression (**D**) in chicken liver. The birds were injected intramuscularly with 0.5, 1.0 and 2.0 mg of 17 β-estradiol (dissolved in olive oil) per kg of body weight, respectively. Vehicle group was injected intramuscularly with the same amount of olive oil; (**E**,**F**) qPCR analysis and Western blot analysis of host gene *SLIT2* mRNA (Panel **E**) and protein (**F**) expression in primary hepatocytes incubated for 24 h with 25, 50, 100 nM 17 β-estradiol, respectively. Vehicle group was treated with 0.1% ethanol; (**G**,**H**) The expression changes of miR-218-5p effected by 17 β-estradiol in the liver tissue measured by qPCR (**G**) and semi-quantitative (**H**); (**I**) Immunoblot analysis of *ELOVL5* expression in liver treated with different concentrations of estrogen in vivo. *SLIT2* and miR-218-5p expression levels are normalized to *β-Actin* and *U6*, respectively. Data represent Mean ± SEM (*n* = 6). * *p* < 0.05; ** *p* < 0.01.

miR-218-5p was found to be encoded from the intron 14 of slit guidance ligand 2 (*SLIT2*) gene in the chicken genome (Figure 5A). To investigate whether miR-218-5p was decreased due to the host gene *SLIT2*, resulting in the upregulation of *ELOVL5* after 20 weeks of age in the liver of laying hens, miR-218-5p and *ELOVL5* mRNA in chicken liver at different developmental stages were detected using qPCR. The results showed that the expression levels of *SLIT2* mRNA increased from 10 to 20 weeks of age while it decreased from 20 to 35 weeks of age. This trend was consistent with expression of miR-218-5p in the liver from 10- to 35-week-old chickens (Figure 5B). The correlation coefficient between miR-218-5p and *SLIT2* mRNA expression in chicken liver at different developmental stages was 0.83 (*p* < 0.05) (data not shown). This result suggests that the expressions of miR-218-5p and *SLIT2* mRNA are co-regulated in chicken liver at different developmental stages. To verify whether the expression of *SLIT2* mRNA was regulated by estrogen, the expression level of *SLIT2* and miR-218-5p were investigated in the livers and primary hepatocytes of chickens treated with 17 β-estradiol. The results showed that the expression of *SLIT2* and miR-218-5p decreased significantly with the increase of estradiol concentration in vitro and in vivo (*p* < 0.01 and *p* < 0.05) (Figure 5C–H). In addition, the *ELOVL5* protein tended to increase in the liver treated with 17 β-estradiol (Figure 5I).

### 2.6. Estrogen Impacts on LCPUFA Synthesis in Chicken Liver

**Figure 6:**
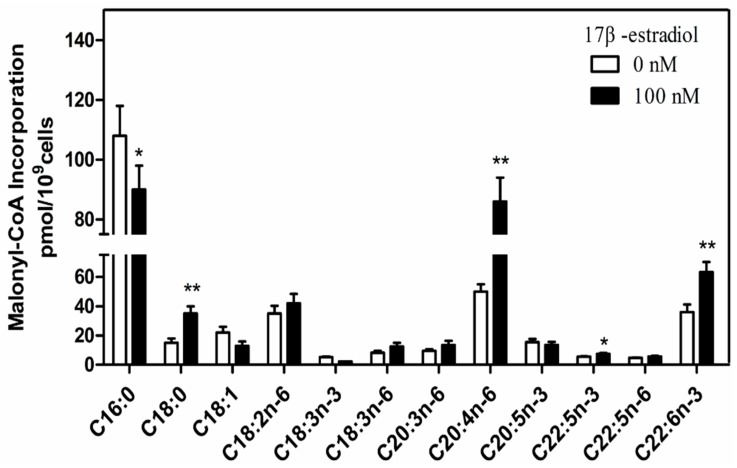
Estrogen enhances hepatic synthesis of LCPUFA. Measurement of fatty acid concentrations in hepatocytes incubated for 24 h with 100 nM 17 β-estradiol, or vehicle (0.1% ethanol). * *p* < 0.05; ** *p* < 0.01.

To further test whether hepatic LCPUFA synthesis was enhanced by estrogen in vitro, fatty acid analysis of chicken primary hepatocytes treated with 17 β-estradiol was performed. Our results revealed an increasing trend in *n*-3 and *n*-6 LCPUFA and a marked increase in C18:0, C20:4*n*-6, C22:6*n*-3 (*p* < 0.01) and C22:5*n*-3 (*p* < 0.05), respectively (Figure 6).

## 3. Discussion

The effect of dietary combination of fatty acids on chicken muscle tissue (*n*-3) long-chain PUFA (LCPUFA) has been investigated in many studies [18,19,20]. The chicken elongase enzymes are unlike any species studied to date because *ELOVL5* can elongate DPA [1,4,6], whereas mammalian *ELOVL5* enzymes are unable to elongate DPA and most fish *ELOVL5* enzymes have low or undetectable activity toward DPA. In other words, chicken could be a dietary source of EPA and DHA instead of, or in addition to fish.

The elongase of ELOVL is rate limiting for long chain fatty acid (LCFA) synthesis [6]. It plays an important role in regulating lipid biosynthesis, fatty acid metabolism and some metabolic diseases. The de novo synthesis of fatty acids (FA) in the liver of poultry plays an important role in the deposition of fatty acids in muscle tissue and yolk. Exogenous nutrients are the main substrates for FA de novo synthesis, and FA synthesis in birds occurs mainly in the liver. Gregory et al. [1] found that the relative abundance of chicken *ELOVL5* is greater than that in rats. In addition, the duck and turkey *ELOVL5* enzyme activities are different from the chicken *ELOVL5*, which can utilize DPA to synthesize 24:5n-3 [4]. A previous study suggested that the duck and turkey *ELOVL5* enzyme activities were different from the chicken *ELOVL5*, which has unique DPA to 24:5n-3 activity [1,4]. Further studies need to be conducted to explore the molecular mechanism behind the ability of chicken *ELOVL5* to elongate DPA to 24:5n-3.

In the present study, we found that *ELOVL5* is highly expressed in the liver and kidney of laying hens, and was significantly increased in peak-laying-period hens (30 weeks old) than in pre-laying hens (20 weeks old). We focused on estrogen, which has a high concentration in laying hen plasma comapred to immature pullets [21]. Moreover, for chicken, the liver is one of the main target organs for estrogen. Further studies focusing on the function of chicken *ELOVL5* gene in the lipid metabolism of the kidney are needed.

Estrogen is vitally important for sexual maturity and the development of the female reproductive system [12,22]. Previous studies suggested that the plasma estrogen in hens reaches peak level just before the onset of the first egg production, then goes down gradually, but still maintains a relatively higher level than that in the immature pullets for a certain period [11,22,23]. The increased estrogen correlates with the initiation of estrogen-dependent gene transcription [24]. Estrogen often plays a physiological role through the estrogen/estrogen responsive element (E2/ER) complex and then interacts with upstream estrogen response element (ERE) in target genes [25,26]. Very low density apolipoprotein II (*ApoVLDLII*), a typical estrogen-dependent gene, is generally used as biological marker to study the mechanism of estrogen. The expression of *ApoVLDLII* in chicken liver and primary hepatocytes were both significantly upregulated after estrogen treatment, which indicated that estrogen treatment was successfully established as a model in this study (Figure S1 and Figure S2). However, in the present study, no putative estrogen receptor binding site was found in the upstream of the *ELOVL5* promoter (data not shown). Our results suggested that estrogen might not be the direct reason for the increased expression of *ELOVL5* in peak-laying period hens.

To further investigate the potential mechanism for up-regulation of *ELOVL5* in the liver of peak-laying period hens, we focused on the post-transcriptional regulators, especially miRNA, which were recently reported to participate in hepatic lipid and fatty acids metabolism [17,27,28]. Our previous research revealed 47 significantly downregulated miRNAs in the liver of peak-laying-period hens. Among them, we found the seed sequences of miR-218-5p, miR-19a-3p, miR-19b-3p, miR-30a-5p, miR-30b-5p, miR-30d-5p, and miR-30e-5p were complementary with the 3′UTR of *ELOVL5* (Figure 3A). The expression level of miR-218-5p was negatively correlated with the mRNA level of *ELOVL5* in the liver at different developmental stages (Pearson correlation *r* = −0.906, *p* = 0.034). The double luciferase reporter assay performed by pcDNA3.1 and psiCheck2 vector system indicated a direct targeting interaction between miR-218-5p and the 3′UTR of *ELOVL5*. Overexpression and knockdown of miR-218-5p in primary hepatocytes significantly up- and down-regulated *ELOVL5* expression at both transcriptional and translational levels. To further investigate whether these miRNA were downregulated by estrogen, we examined the expression level of these miRNA after treated with 17 β-estradiol in vitro. The expression level of miR-218-5p, miR-30-5p and miR-19-3p was downregulated by estrogen. Thus, our results suggested that estrogen can function as a regulator which suppresses miRNA, thereby protecting *ELOVL5* transcripts from miRNA-mediated suppression in the liver of laying hens.

## 4. Materials and Methods

### 4.1. Ethics Statement

All animal experiments were performed in accordance with protocols and guidelines approved by the Institutional Animal Care and Use Committee (IACUC) of Henan Agricultural University, China (Permit Number: 11-0085; Date: 06-2011).

### 4.2. Animals and Tissue Collection

The experimental animals used in this study were Lushi green-shell laying hens obtained from the Animal Center of Henan Agricultural University. All birds were raised in the same environmental conditions with ad libitum access to food and water. Forty-eight healthy individuals were sampled randomly and then slaughtered at the stage of 1, 10, 15, 20, 30, 35 and 36–50 weeks of age. Tissues including liver, heart, lung, kidney, duodenum, ovary, pectorals and abdominal fat were immediately collected, snap-frozen in liquid nitrogen, and stored at −80 °C until RNA extraction. Healthy female individuals (*n* = 32) were sampled randomly at 10 weeks of age and then divided into four groups. The first group was the control group, the other three groups were experimental groups. 17 β-estradiol was used at the previously recommended concentrations [22,29]. After three days of conventional feeding, experimental groups were injected intramuscularly with 0.5, 1.0, and 2.0 mg of 17 β-estradiol (E2) (Sigma, St. Louis, MO, USA) (dissolved in olive oil)/kg of body weight, respectively. The birds in control group were injected intramuscularly with olive oil alone. All the birds were killed after 12 h of treatment. Livers were collected and stored as mentioned above.

### 4.3. Cell Culture

Hepatocytes were isolated from chicken embryonic livers using the method described by Fischer and Marks [30], with some modifications. In brief, livers were removed from 18 or 19-day-old embryonic chickens in a sterile environment, manually minced washed with d-Hanks solution (Solarbio, Beijing, China). Then digested by collagenase type II (Solarbio) with gentle shaking at 37 °C for 30 min. Dispersed livers were filtered through a 100-, 200- and 500-mesh sieve. After washing and centrifugation, hepatocytes were obtained using the discontinuous density Percoll gradient centrifugation technique; the filtered cell suspension was resuspended in William’s E medium (Sigma), supplemented with 10% fetal calf serum (Gibco, Australia), 0.22% NaHCO_3_, 100 mg/mL streptomycin and 100 U/mL penicillin, and counted using a Luna automated cell counter (Biosystems L10001, Anyang-si, Korea). Hepatcoytes were adjusted to a cell density to 5 × 10^5^ cells/mL. Cell number and viability were verified by the trypan blue exclusion test. Cells were cultured in 6-well plates in William’s E medium at 37 °C with 5% CO_2_ in a humidified incubator. Primary hepatocytes were cultured for approximately 24 h (the cells reached 90% confluence), the cell culture medium was replaced by serum-free William’s E medium with streptomycin and penicillin, and incubated for 6 h. The chicken fibroblast cell line (DF-1) was maintained in Dulbecco’s modified Eagle’s (DMEM) medium with 10% fetal bovine serum (FBS) (Gibco), 100 mg/mL streptomycin and 100 U/mL penicillin at 37 °C with 5% CO_2_ in a humidified incubator.

### 4.4. 17 β-Estradiol Treatment and Transfection of Chicken Hepatocytes

To determine the effect of estrogen on the expression of genes in hepatocytes, the cells were divided into four groups, with six replicates in each group, and treated with 25, 50 and 100 nM of 17 β-estradiol (dissolved in ethanol), respectively. The control group was treated with ethanol alone at a final concentration of 0.1%. To overexpress or knockdown miR-218-5p, chicken hepatocytes were seeded at a density of 1.5 × 105 cells/mL in 6-well plates for 24 h and transfected with miR-218-5p mimics (50 nM) or a negative control (50 nM), miR-218-5p Inhibitors (50 nM) or Inhibitors NC (50 nM) using the TurboFect Transfection Reagent (Thermo Scientific, Waltham, MA, USA) according to the manufacturer’s protocol. Chicken hepatocytes were washed with fresh D-Hanks solution after 12 h incubation, and collected with trizol reagent (Takara, Dalian, China), stored at −80 °C until use.

### 4.5. miRNA Sequencing Data and Bioinformatics Analysis

The miRNA sequencing data from pre- (20-week old) and peak-laying hens’ (30-week old) liver tissue from Lushi green-shell laying hens was obtained from our previous study [17]. The raw data are available from the NCBI GEO database (GSE74242). Differentially expressed miRNA with |log2FC| ≥ 1.5 and with false discovery rate (FDR) < 0.05 were selected as candidate miRNA. The 3′UTR sequences of gallus *ELOVL5* were downloaded from the 3′UTR database (http://utrdb.ba.itb.cnr.it/). The miRNA target prediction software miRDB (http://mirdb.org/miRDB), Targetscan 7.1 (http://www.targetscan.org/) and PicTar (http://pictar.org/) were employed to predict miRNA binding sites in chicken *ELOVL5* 3′UTRs. The DIANA-miRPath v3.0 tool [31] (http://snf-515788.vm.okeanos.grnet.gr/) was used for the miRNA target pathway analyses.

### 4.6. Vector Construction

The 3′UTR of *ELOVL5* containing a miR-218-5p binding site was amplified from chicken genome DNA by PCR, and cloned into the XhoI–NotI site of the psiCHECK-2 vector (Promega, Maddison, WI, USA), named psiCHECK2-ELOVL5-3′UTR-WT. A mutant 3′UTR of *ELOVL5* reporter was generated by mutating the seed region of the miR-218-5p binding sites by overlapping extension PCR, named psiCHECK2-ELOVL5-3′UTR-Mut (Figure S4). The DNA sequence encoding the miR-218-5p precursor was PCR amplified from chicken genomic DNA. And the PCR result was cloned into the XhoI–XbaI site of the pcDNA3.1-EGFP vector (Invitrogen, Carlsbad, CA, USA). To confirm the constructs, bacteria liquid PCR and sequencing (Sangon Biotech, Shanghai, China) were performed. Plasmid DNA was extracted and purified by using the EndoFree Maxi Plasmid Kit (TIANGEN, Beijing, China). Lipofectamine 2000 (Thermo, Shanghai, China) was used for plasmid transfection.

### 4.7. Luciferase Reporter Assay

To determine if miR-218-5p targets the *ELOVL5* 3′UTR, DF1 cells were seeded in 12-well plates in triplicate and transfected with a wild-type or mutant construct in serum-free DMEM medium. After 6 h of transfection, the medium was changed. Cells were harvested and lysed 48 h after transfection. Luciferase activity was measured using the Dual-Luciferase^®^ Report Assay System (Promega, Maddison, WI, USA) on a Fluoroskan Ascent FL instrument (Thermo Fisher Scientifc, Shanghai, China). Renilla luciferase activity was normalized to Firefly luciferase activity. And this transfection experiment was performed in triplicate wells and repeated at least three times.

### 4.8. RNA Isolation and Quantitative Real-Time PCR (qPCR)

Total RNA for tissues and chicken primary hepatocytes was extracted with Trizol reagent following the manufacturer’s instructions. RNA concentrations and integrity were determined by NanoDrop 2000 spectrophotometry (Thermo Scientific, Wilmington, DE, USA) and standard denaturing agarose gel electrophoresis, respectively. RNA was reverse-transcribed using the PrimeScript™ RT reagent Kit with gDNA Eraser (TaKaRa) according to the manufacturer’s instructions. The cDNA was stored at −20 °C until use. The primers for qPCR were designed by using online software Primer3plus (http://primer3.sourceforge.net/webif.php) and synthesized by sangon biotech Co., Ltd. (Shanghai, China) (Table S2). The expression of miRNA was detected by stem-loop real-time qPCR. The stem-loop primers used for the qPCR, miRNA mimics, miRNA mimics NC, miRNA inhibitor and inhibitor NC were purchased from GenePharma Co., Ltd. (Shanghai, China). qPCR was performed in triplicate using the SYBR^®^ Premix Ex Taq™ II kit (Takara) on a LightCycler^®^ 96 Real-Time PCR system (Roche, Basel, Switzerland) according to the method of Nolan [32]. Each reaction contained 1 µL cDNA, 5 µL 2× SYBR^®^Premix Ex Taq™ II (TliRNaseH Plus) (TaKaRa), 0.5 µL each of forward and reverse primers (10 µM), and 3 µL deionized water. The qPCR amplification procedure for mRNA was as follows: 95 °C for 3 min; 35 cycles of 95 °C for 30 s, 60 °C for 30 s, 72 °C for 20 s, and an extension for 10 min at 72 °C. And the qPCR amplification procedure for miRNA was as follows: 95 °C for 3 min; 40 cycles of 95 °C for 12 s, 60 °C for 40 s, 72 °C for 30 s, and an extension for 10 min at 72 °C. The mRNA expression levels were normalized to *β-Actin*. Chicken small nuclear RNA *U6* was used as the internal control for miRNA. The 2 ^–ΔΔ*C*t^ method was used to determine the relative mRNA and miRNA abundance [33]. The sequences of primers for this study are listed in Table S2.

### 4.9. Western Blot

Total protein was extracted from liver tissue and cells using a RIPA lysis buffer supplemented with phenylmethyl sulfonyl fluoride (Servicebio, Wuhan, China) (100:1). Protein was separated on 10% SDS-PAGE gels. Separated protein was then transferred to polyvinylidene difluoride (PVDF) membranes (ISEQ00010, Millipore, Danvers, MA, USA) and membranes were then blocked with nonfat milk (5%) for 1 h. The membranes were washed with PBST three times (5 min/time) and incubated with the primary antibodies (Abcam) at 4 °C for overnight. Then the membranes were washed three times using PBST, and incubated with secondary antibody conjugated with HRP (GB23303, Servicebio, China) for one hour at room temperature. Signals were enhanced by ECL Plus (Solarbio) and images were captured and analyzed by Photoshop cs 6 and AlphaView 3.0 (Alpha Innotech, San Jose, CA, USA). *β-Actin* was used as an internal control.

### 4.10. Fatty Acid Extraction and Analysis

The primary hepatocytes were seeded in 90-mm culture dish (Corning Inc., Corning City, NY, USA) and incubated with 100 nM of 17 β-estradiol for 12 h. The control group was treated with ethanol at a final concentration of 0.1%. Two groups with three repeats in each group. The FA extraction of hepatocytes was analyzed following the method described previously [34,35,36,37], with some modifications. Briefly, the rinsed cell pellet was extracted with 2 mL of sulfuric acid/methanol (1:40, *vol*/*vol*), ultrasound was used to disrupt cells, then in a water bath at 80 °C for 60 min. After saponification and methylation, the reaction solution was store at room temperature for 15 min, and the total cell lipids were extracted containing 1 mL N-Hexane, 2 mL 0.1 M HCl solution. FAME were then analyzed by GC using an Agilent 7890 A chromatograph equipped with an FID and a 30-m fused-silica capillary column (0.32 mm, i.d.) coated with 0.25 µm Omegawax (Supleco, Bellefonte, PA, USA). Helium was used as carrier gas at a column flow rate of 1 mL/min. The inlet split ratio was set at 10:1.The oven temperature programming was held at 40 °C for 2 min before being raised to 180 °C at 8 °C/min for 2 min and finally to 240 °C at 3 °C/min for up to 10 min. The injector and detector temperatures were 240 °C. The FAME were identified by comparison of their retention times with authentic standards.

### 4.11. Statistical Analyses

Statistical analyses of the qPCR results were carried out using SPSS version 19.0 (IBM , Chicago, IL, USA). One-way ANOVA and repeated measures ANOVA were used for statistical analysis of relative expression levels, followed by Dunnett’s test. Graphics were drawn using GraphPad Prism 5 software (San Diego, CA, USA). The results were presented as Mean ± SEM of three replicates, *p* < 0.05 and *p* < 0.01 were considered statistically significant and highly significant, respectively.

## 5. Conclusions

**Figure 7:**
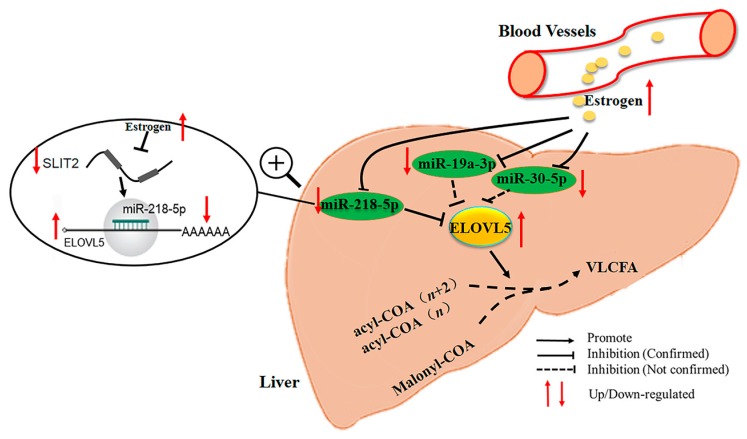
The pathway of estrogen regulation of hepatic synthesis of LCPUFA at post-transcriptional level in laying hens. Briefly, with the arrival of sexual maturity, the body of blood estrogen content increased rapidly; estrogen eliminates the suppressive effect of miRNA on the target gene *ELOVL5*. Interestingly, estrogen suppresses the host *SLIT2* gene thus decreasing the expression of intronic miR-218-5p to promote hepatic synthesis of long-chain polyunsaturated fatty acids in the liver of laying hens. VLCFA, very long chain fatty acid.

In summary, our study suggests important roles of hormones and miRNA in the hepatic synthesis of LCPUFA, indicating that the increased secretion of estrogen promotes hepatic LCPUFA synthesis by upregulation of ELOVL5 expression in the laying period. These data provide a novel model in the poultry industry for the use of estrogen as an inducer of ELOVL5 expression to enhance hepatic n-3 and n-6 LCPUFA synthesis at the post-transcriptional level (Figure 7).

## Supplementary Materials

Supplementary materials can be found at www.mdpi.com/1422-0067/18/7/1405/s1.

## Abbreviations

PUFA	Polyunsaturated fatty acids
FA	Fatty acids
EPA	Eicosapentaenoic acid
DPA	Docosapentaenoic acid
DHA	Docosahexaenoic acid
ELOVL5	Elongase-5
SLIT2	Slit guidance ligand 2
ApoVLDLII	Very low density apolipoprotein II
UTR	Untranslated regions
WT	Wild-type
Mut	Mutant-type
E2	Estradiol
ERE	Estrogen responsive element
E2/ER complex	Estrogen/estrogen responsive element complex
