# Supplementary material for: Estrogen Promotes Hepatic Synthesis of Long-Chain Polyunsaturated Fatty Acids by Regulating ELOVL5 at Post-Transcriptional Level in Laying Hens
Source: Int J Mol Sci. 2017 Jun 30;18(7):1405. doi: 10.3390/ijms18071405 (PMC5535898; doi:10.3390/ijms18071405)
Supplement: Supplementary file 1 [file ijms-18-01405-s001.zip › ijms-204176-suppl.pdf]

Supplementary material

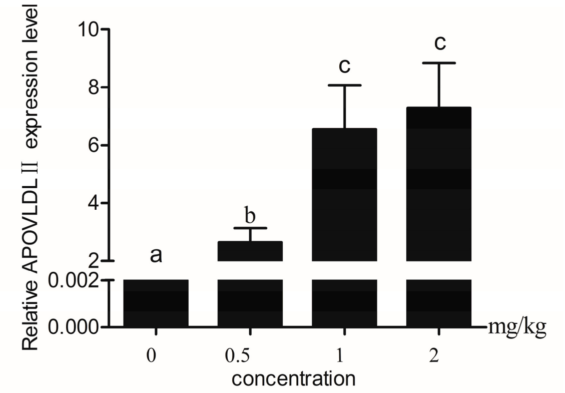

Figure S1. Expression level of *ApoVLDLII* mRNA in the liver of laying hens. The birds were injected intramuscularly with 0.5, 1, and 2 mg 17 β-estradiol (dissolved in olive oil) per kg of body weight, or with the vehicle (the same amount of solvent olive oil). *β-Actin* was used as the referenced gene to estimate mRNA. Data are presented as Mean ± SEM ($n = 6$ for each group). Values with different superscripts indicate statistical difference ($p < 0.05$).

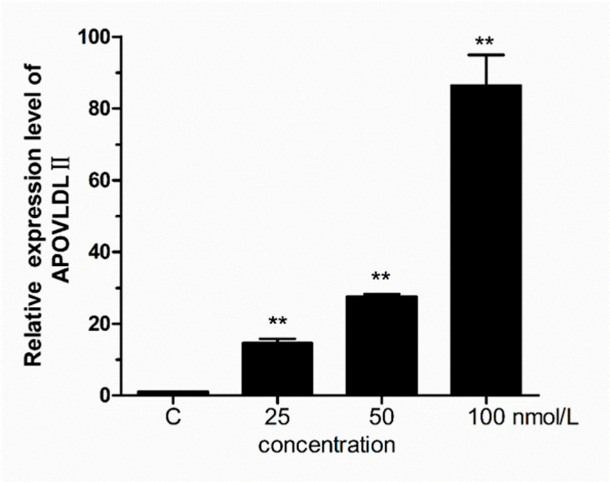

Figure S2. Expression level of *ApoVLDLII* mRNA in chicken primary hepatocytes. The cells were incubated with 25, 50, and 100 nM of 17 β-estradiol, or vehicle (0.1% ethanol). β-Actin was used as the reference gene to estimate mRNA. Data are presented as Mean ± SEM ($n = 6$ for each group). * $p < 0.05$, ** $p < 0.01$.

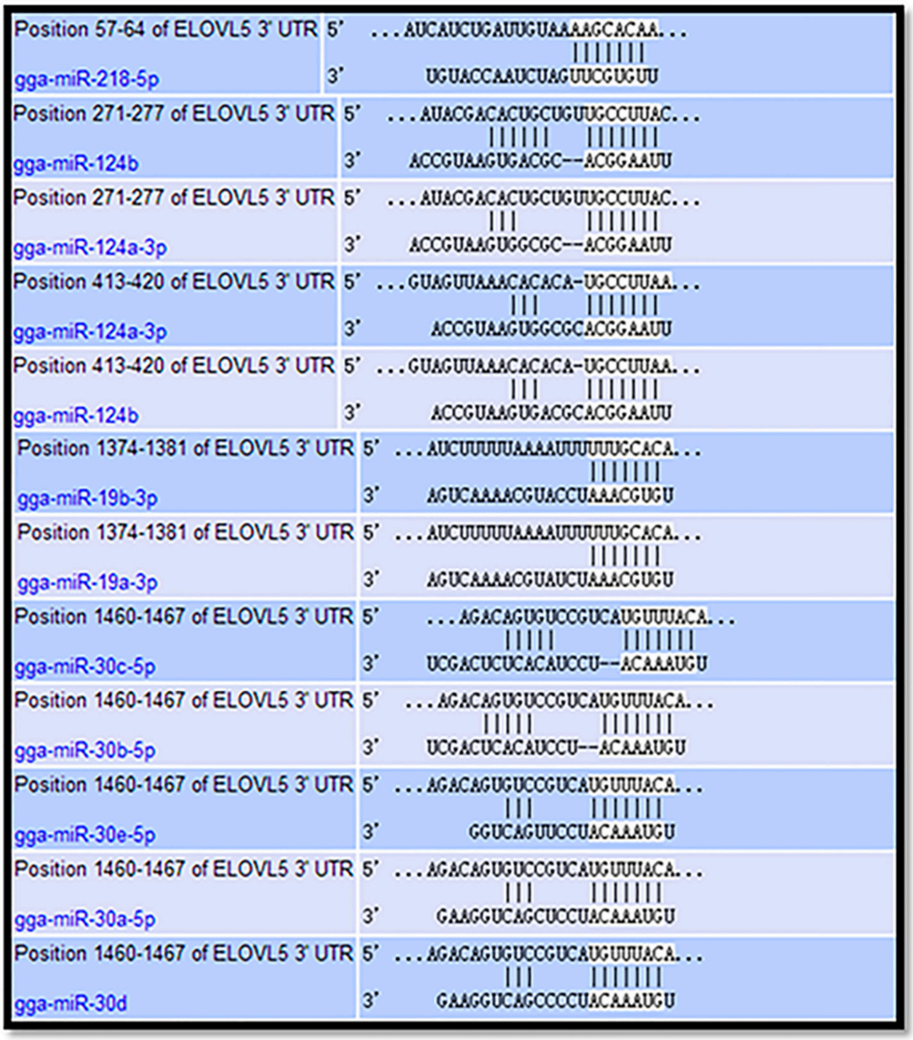

Figure S3. The binding sites in the 3′UTRs of target gene *ELOVL5* complemented with seed regions of miRNAs.

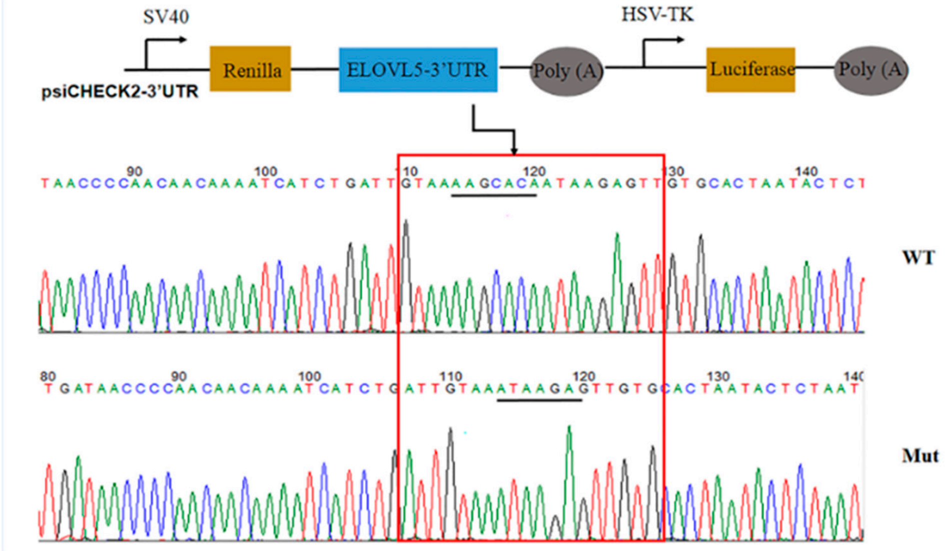

Figure S4. Construction and verification of plasmids. The 3′UTRs of target gene *ELOVL5* were cloned into psiCHECK-2 Dual Luciferase Reporter vectors for validation in vitro. WT, Wild type vector; Mut, Mutated vector.

Table S1. Downregulated miRNAs in the liver of peak-laying period hens.

miRNA	ǀFold Change (L20/L30)ǀ	FDR
miR-146b-5p	8.5	0
miR-24-3p	7.4	1.696E-27
miR-146a-5p	6.0	3.81E-122
miR-221-5p	5.8	0.0244488
miR-7b	5.4	0.0001006
miR-147	5.1	0.0415756
miR-106-5p	5.0	0.088337
miR-20a-5p	4.6	4.977E-19
miR-140-5p	4.6	1.655E-08
**miR-218-5p**	**4.3**	**0.0385142**
**miR-19b-3p**	**4.3**	**3.687E-21**
miR-33-3p	4.2	6.707E-06
miR-16c-5p	4.0	0.000481
miR-221-3p	4.0	9.691E-12
**miR-19a-3p**	**3.9**	**6.599E-05**
miR-16-5p	3.8	1.116E-57
miR-146c-5p	3.7	0
miR-223	3.6	6.169E-06
miR-451	3.6	2.422E-62
miR-126-3p	3.6	3.22E-115
miR-10a-5p	3.5	0
miR-142-3p	3.5	3.956E-06
miR-126-5p	3.4	0
miR-17-5p	3.4	1.676E-09
miR-2188-5p	3.3	1.092E-13
miR-181a-3p	3.3	0.0106489
miR-23b-3p	3.3	0.0388315
miR-155	3.2	0.0428774
miR-193b-3p	3.2	2.564E-19
miR-200a-3p	3.2	6.113E-06
miR-21-3p	3.0	0.0094551
miR-122-3p	3.0	9.133E-18
miR-215-5p	2.9	0.0104972
**miR-30a-5p**	**1.7**	**0.000341357**
miR-34a-5p	2.8	0.0001375
miR-21-5p	2.5	0
miR-214	2.5	0.0399318
**miR-30e-5p**	**2.5**	**0.0020146**
miR-140-3p	2.4	1.579E-37
miR-199-5p	2.4	5.453E-06
**miR-30b-5p**	**2.4**	**0.0014809**
miR-29a-3p	2.3	0.0165375
miR-142-5p	2.3	2.285E-21
miR-122-5p	2.2	4.38E-207
miR-130b-3p	2.1	0.0461745
miR-101-3p	2.1	1.257E-16

Note: miRNA with binding sites at the 3′UTR of *ELOVL5* are in bold. L20/L30, miRNA expression level (Tpm) of 20 and 30-week-old Lushi chicken. FDR, False Discovery Rate.

Table S2. List of cloning and qPCR primers used in the present study.

Gene name	Primer sequences(5′-3′)	Product size (bp)	Tann (℃)	Purpose
ELOVL5-F	ATTGGGTGCCTTGTGGTCA	180	60	qPCR
ELOVL5-R	AGCTGGTCTGGAAGATTGTCA			
APVLDLII-F	CAATGAAACGGCTAGACTCA	108	60	qPCR
APVLDLII-R	AACACCGACTTTTCTTCCAA			
SLIT2-F	CGTCTGGCAAACAAAAGGAT	191	60	qPCR
SLIT2-R	CTGTGGGATGTGATCAGGAA			
β-actin-F	GGACACCAAGCTCAGAGACT	112	60	qPCR
β-actin-R	GGACACCAAGCTCAGAGACT			
Pre-miR-218-1-F	CCGCTCGAGTCCCTCACTGTTACCCTTGG	174	60	Cloning
Pre-miR-218-1-R	TGCTCTAGATGACTTAAGCCTCAGCAGCA			
ELOVL5-F	CCGCTCGAGTGCCTGAAAGACCAAACTGA	230	59	Cloning
ELOVL5-R	ATTTGCGGCCGCGGTCAGTGACAGCCCAGAAT			
ELOVL5-Fm	ACAAAATCATCTGATTGTAAATAAGAGTTGTGCACTAATA	230	59	Cloning
ELOVL5-Rm	TATTAGTGCACAACTCTTATTTACAATCAGATGATTTTGT			

Note: F, forward primer for qPCR; R, reverse primer for qPCR and cloning. Fm, forward primer for cloning mut vector; Rm, reverse primer for cloning mut vector. Tann, annealing temperature for PCR program.
